# Nationwide population-based incidence of cancer among patients with HIV/AIDS in South Korea

**DOI:** 10.1038/s41598-022-14170-5

**Published:** 2022-06-15

**Authors:** Soon Ok Lee, Jeong Eun Lee, Shinwon Lee, Sun Hee Lee, Jin Suk Kang, Hyunjin Son, Hyungi Lee, Jinmi Kim

**Affiliations:** 1 Division of Infectious Diseases, Department of Internal Medicine, Pusan National University Hospital, Pusan National University School of Medicine and Medical Research Institute, 179 Gudeok-ro, Seo-gu, Busan, 49241 Korea; 2 Department of Internal Medicine, Inje University School of Medicine, Busan Paik Hospital, Busan, Korea; 3 Department of Prevention Medicine, Donga University School of Medicine, Donga University Hospital, Busan, Korea; 4 Department of Statistics, Biomedical Institution, Pusan National University Hospital, Busan, Korea

**Keywords:** Infectious diseases, HIV infections

## Abstract

Cancers are the leading cause of death among people living with HIV/AIDS (PLWHA); however, nationwide studies on cancer incidence are limited. We aimed to determine the trends in the incidence rates of AIDS-defining cancers (ADCs) and non-AIDS-defining cancers (NADCs) among Korean PLWHA. Data from the National Health Insurance Sharing Service from 2004 to 2017 were collected. Age- and sex-adjusted standardized incidence ratios (SIRs) for various cancer types relative to the general population were calculated. Of the 11,737 PLWHA followed-up for 65,052 person-years (PYs), 445 (ADCs, 130 and NADCs, 298) developed cancer. The incidence rate of ADCs decreased, whereas that of NADCs remained unchanged. PLWHA were at an increased risk of ADCs (SIR: 12.6, 95% CI: 10.6–15.0), including Kaposi’s sarcoma, non-Hodgkin’s lymphoma, and cervical cancer, and some NADCs, including anal cancer, lung cancer, liver cancer, and oropharyngeal cancer. Of the 396 patients who received antiretroviral therapy (ART), 215 with optimal adherence had lower incidence rates for ADCs and NADCs than those with non-optimal adherence. The 5-year survival rate of PLWHA with NADCs was 57.8%. Close surveillance and routine screening of cancers and improvement in ART adherence are required to improve the clinical outcomes of PLWHA.

## Introduction

Since the introduction of highly active antiretroviral therapy (HAART), the life expectancy of PLWHA has dramatically increased, and consequently, prolonged survival time has led to variations in the death causes^1–5^. Non-AIDS-related deaths caused by chronic illnesses related to aging, such as cancer and cardiovascular disease, are increasing in most developed countries^6–8^. A study conducted in Japan from 2005 to 2014 revealed that cancer is the leading cause of death, accounting for 47% of all cases among PLWHA^7^.

PLWHA have an elevated cancer risk due to non-infectious causes, such as smoking and alcohol consumption, and HIV-related immunosuppression, which impairs the control of oncogenic viruses. With the advent of HAART, the incidence rates of AIDS-defining cancers (ADCs) such as Kaposi’s sarcoma, non-Hodgkin’s lymphoma (NHL), and cervical cancer decreased, whereas the burden of non-AIDS-defining cancers (NADCs) increased. In particular, virus-related NADCs, such as Hodgkin lymphoma (Epstein Barr Virus-related), liver cancer (Hepatitis B and C virus), anogenital cancer (human papillomavirus, HPV), and oropharyngeal cancer (HPV), are considerably more prevalent in PLWHA than in the general population. PLWHA are diagnosed with cancer in advanced stages and have a higher cancer-specific mortality rate than the general population after cancer diagnosis^9–11^. Therefore, for long-term survival of PLWHA, early detection and treatment of cancer through active screening is important. Most of these studies have been conducted in Western countries, and studies in Asian countries have mostly focused on Taiwan, where the National Health Insurance programs and databases have been established.

Three studies were conducted in Korea, of which two studies published in 2006 and 2009, reported that there was no decrease in the ADC incidence nor a relative increase in NADC incidence, and the incidence pattern of NADC varied by institutions^12,13^. According to a study published in 2017 in Korea, there was an increase in NADC incidence compared to ADC incidence, without statistically significant difference^14^. However, the sample size was small, and research in a single institution is not enough to represent the incidence of cancer in PLWHA in Korea. To establish an appropriate screening plan for the early detection and treatment of cancer, it is important to obtain information regarding cancer types and trends among Korean PLWHA. Therefore, this study aimed to estimate and compare the risk of cancer incidence between the general population and Korean PLWHA and to investigate the recent trends in cancer among Korean PLWHA from 2004 to 2017, using a nationwide population-based database.

## Methods

### Study design and database

We conducted a nationwide retrospective cohort study using the 2002–2017 data from the Korea National Health Insurance Service (NHIS) database. This database includes data on patients’ sociodemographics, healthcare provider characteristics, diagnoses based on the International Classification of Disease and Related Health Problems 10th revision (ICD-10), procedures or operations, pharmacy dispensing claims, and medical care costs^15,16^. The NHIS is a mandatory public medical insurance system operated by the South Korean government. Almost the entire Korean population is covered by either the NHI (97.2%) or medical aid (2.8%)^17^. As the NHIS database represents the entire Korean population, it can therefore be used in nationwide population-based studies of various diseases, including HIV/AIDS^18^.

### Patient identification

Data from all 38,205 patients who had HIV/AIDS-related codes (ICD-10 codes B20–B24, O987, Z21, R75 and V103) between 2002 and 2017 were extracted from the database (Supplementary Fig. 1). Foreigners (*n* = 920), patients with unknown age and sex (*n* = 142), and patients who did not undergo any follow-up tests (HIV RNA test, HIV drug resistance mutation sequencing, or T cell subset analysis; *n* = 22,863) were excluded. In suspected HIV cases, that is patients who were surveillance tested, physicians sometimes used the above-mentioned ICD-10 codes in their medical records and insurance claims until they perform a confirmatory test. Therefore, we excluded patients with less than three outpatient visits or no h ospitalization to avoid false-positive cases^19^. To identify HIV incidence cases, 1224 patients that were considered as prevalance cases, having HIV-related codes, were deselected between 2002 and 2003. We excluded those patients in whom we could not pinpoint the time they were given their HIV diagnosis. This was done to observe all patients from the time of diagnosis of HIV to the time of cancer for the purpose of the study. After excluding 54 patients aged < 15 years from this cohort, we determined that from 2004 to 2017, there were 12,515 patients with HIV/AIDS aged ≥ 15 years in Korea.

The government of the Republic of Korea initiated a registration program for rare and intractable diseases (RID) and expanded benefit coverage for patients with HIV/AIDS from 2004. Patients with HIV/AIDS registered in this program were eligible for up to 90% co-payment reduction. Because physicians only registered patients identified by confirmatory test results, data regarding RIDs are verified and reliable. Most patients register in the RID program to reduce medical expenses; however, some patients may refuse registration because they are reluctant to disclose their health status. Thus, we defined HIV infection when a patient met the above criteria rather than defining it using the RID code. We investigated the match of patients with the RID code (V103) among those who met the above criteria to check the diagnostic accuracy. Of the 12,515 patients mentioned above, 12,068 (96.4%) met the RID code (V103).

Of the 12,515 patients, 448 who were diagnosed with cancer before the diagnosis of HIV were excluded to avoid prevalent cancer cases. Further, 290 patients who were diagnosed with cancer within the first 90 days after the diagnosis of HIV, were considered prevalent cases and were not included in our study^20,21^. The final number of patients was 11,737. The first date of HIV-related code registration in the database was regarded as the date of diagnosis. Patients were observed from the day of first hospital visit to the date of cancer diagnosis, death, or December 31, 2017.

### Operational definitions

Cancer was first identified based on the ICD-10 codes, including C00–C99, and the types were subdivided according to the cancer site. Cancer was defined as the presence of the same C code more than three times within a year or inpatient hospitalization with a C code^22^. In Korea, a person diagnosed with cancer is registered with the National Cancer Registry with a specific code (called the C code) to indicate cancer diagnosis and to provide special insurance benefits^22^. Therefore, cancer diagnoses based on claims are considered reliable. If a patient was diagnosed with multiple cancers, only the first cancer was considered.

According to the CDC classification criteria, NHL, Kaposi’s sarcoma, and cervical cancer were classified as AIDS-defining cancers (ADCs), whereas all other types of cancer were classified as non-AIDS-defining cancers (NADCs)^23^. To evaluate cancer trends, we divided the overall observation period into three: period 1, 2004–2008; period 2, 2009–2013; and period 3, 2014–2017.

Comorbidities were defined based on information obtained prior to or during HIV diagnosis. Comorbidities were evaluated using Charlson’s comorbidity index (CCI). Because HIV/AIDS and cancer were the variables of interest, we used the modified CCI to exclude these two diseases from the list of comorbidities. Covariates and ICD-10 codes used in the study included the code for chronic hepatitis B virus (HBV) infection (B180, B181) and chronic hepatitis C virus (HCV) infection (B182).

AIDS-defining illnesses were defined according to the 1993 definition of the Centers for Disease Control and Prevention^23^ (Supplementary Table S2). AIDS-defining cancer was excluded because cancer was a variable of interest in our study. Patients requiring prophylactic antibiotics included those with a prescription of a prophylactic dose of oral trimethoprim-sulfamethoxazole or oral dapsone^24^. We regarded individuals requiring prophylactic antibiotics as having a nadir CD4 + T-cell count < 200 cells/µL.

Adherence was evaluated using the medication possession ratio (MPR), calculated as the number of days for which ART prescription was filled divided by the patient’s total follow-up time in days since ART initiation until 31 December 2017, cancer development, or death. To assess the association between cancer risk and ART adherence, we calculated standardized incidence ratios (SIRs) separately for individuals with optimal adherence. Optimal adherence to ART was defined as an MPR ≥ 95%. In the ART group, those who received only one ART regimen or two nucleoside reverse transcriptase inhibitors were excluded because they had possibly received post-exposure prophylaxis, pre-exposure prophylaxis, or treatment for other diseases^25^.

### Statistical analysis

The SAS Enterprise Guide (SAS Institute, Inc.) was used for all statistical analyses. Categorical variables were compared using Pearson’s *χ*^2^ test or Fisher’s exact test, whereas non-categorical variables were tested using the Mann–Whitney U test or Kruskal–Wallis test. Cancer incidence among PLWHA was compared with that in the general Korean population between 2004 and 2017 from the Korea Central Cancer Registry (KCCR) using SIRs obtained by indirect standardization^26^. We calculated the ratio of observed to expected cancers in overall and site-specific cancers among PLWHA. The expected number of cancers was calculated by multiplying the age-specific cancer incidence rate in the general population for 2004–2017 and the number of person-years of PLWHA, and 95% confidence intervals (CIs) were calculated using the Poisson distribution. In addition, we compared the SIRs for optimal adherence using the Poisson regression. Kaplan–Meier curves were used to estimate the overall survival during the study period. All tests of significance were two-tailed, and statistical significance was set at *P* < 0.05.

### Ethics statement

The study protocol was reviewed and approved by the Institutional Review Board (IRB) of the Pusan National University Hospital (approval number 1904–009-078). Our research was performed in accordance with the relevant guidelines and regulations. Informed consent was waived by the IRB of the Pusan National University Hospital because patient information was anonymized.

## Results

### Baseline characteristics

In total, 11,737 patients with PLWHA were followed up for 65,051.53 person-years (median: 4.8 years; range: 2.0–8.6). The total study cohort comprised of 10,911 men with a total follow-up of 60,115.89 person-years and 862 women with 4935.64 person-years. he clinical characteristics of the study population are summarized in Table 1. The mean age at diagnosis was 38.98 ± 13.08 years. There were 93% male patients, 50.8% who required prophylactic antibiotics and 94.3% who received ART.

**Table 1:** Baseline characteristics of PLWHA in the study population (*N* = 11,737).

Characteristics	Total (*N* = 11,737)	With cancer (*n* = 445)	Without cancer (*n* = 11,292)	*P*
**Sex**				0.147
Male	10,911 (93.0)	406 (91.2)	10,505 (93.0)	
Female	826 (7.0)	39 (8.8)	787 (7.0)	
Age, years, at HIV diagnosis*	38.98 ± 13.08	45.97 ± 13.01	38.7 ± 13.01	< 0.001
15–34	4909 (41.8)	90 (20.2)	4819 (42.7)	
35–64	6374 (54.3)	315 (70.8)	6059 (53.7)	
≥ 65	454 (3.9)	40 (9.0)	414 (3.7)	
**Year of entry**				< 0.001
2004–2008	3422 (29.2)	237 (53.3)	3185 (28.2)	
2009–2013	4213 (35.9)	160 (36.0)	4053 (35.9)	
2014–2017	4102 (35.0)	48 (10.8)	4054 (35.9)	
Chronic hepatitis B at and before HIV diagnosis*	692 (5.9)	41 (9.2)	651 (5.8)	0.003
Chronic hepatitis C at and before HIV diagnosis*	797 (6.8)	45 (10.1)	752 (6.7)	0.005
AIDS defining illness	5338 (45.5)	289 (64.9)	5049 (44.7)	< 0.001
Requiring prophylactic antibiotics (CD4 < 200)	5958 (50.8)	273 (61.4)	5685 (50.4)	< 0.001
On antiretroviral therapy	11,066 (94.3)	396 (89.0)	10,670 (94.5)	< 0.001
Optimal ART adherence (MPR > 95%)	7779 (66.3)	215 (48.3)	7564 (67.1)	< 0.001
Modified Charlson’s comorbidity score at and before HIV diagnosis*				0.724
0	2680 (22.8)	105 (23.6)	2575 (22.8)	
1–5	8746 (74.5)	326 (73.3)	8420 (74.6)	
≥ 6	311 (2.7)	14 (3.2)	297 (2.6)	
Median follow-up time, years	4.78 (2.00–8.58)	3.72 (1.49–6.53)	4.84 (2.03–8.68)	< 0.001
Financial status at and before HIV diagnosis*				0.380
NHI	10,687 (91.1)	400 (89.9)	10,287 (91.1)	
National Medical Aid	1050 (9.0)	45 (10.1)	1005 (8.9)	

* The date of HIV diagnosis was defined as the date on which HIV-related code was registered in the database.

The median age of patients with ADCs was 43 years (interquartile range [IQR]: 36–52), and that of those with NADCs was 53 years (IQR: 43–62) (*p* < 0.001). The prevalence of prophylactic antibiotic use was higher in the ADC group (ADC: 72.3% vs. NADC: 56.8%, *p* = 0.003). Optimal ART adherence was higher in the NADC group, albeit not statistically significant (ADC: 42.3% *vs.* NADC: 50.8%, *p* = 0.103) (Table 2).

**Table 2:** Clinical characteristics of malignancy patients among PLWHA (n = 445).

Characteristics	Total (*n* = 445)	ADC (*n* = 130)	NADC (*n* = 315)	*P*
**Sex**	406 (91.2)	116 (89.2)	290 (92.1)	0.337
Male				
Female				
Age at cancer diagnosis (IQR)	50 (41, 59)	43 (36, 52)	53 (43, 62)	< 0.001
Age at HIV diagnosis* (IQR)	46 (37–55)	40 (32–49)	49 (39–57)	< 0.001
Age at HIV diagnosis*	45.97 ± 13.01	40.9 ± 11.78	48.06 ± 12.34	< 0.001
15–34	90 (20.22)	42 (32.31)	48 (15.24)	
35–64	315 (70.79)	84 (64.62)	231 (73.33)	
≥ 65	40 (8.99)	4 (3.08)	36 (11.43)	
**Year of entry**				0.905
2005–2008	237 (53.3)	70 (53.9)	167 (53.0)	
2009–2012	160 (36.0)	45 (34.6)	115 (36.5)	
2013–2016	48 (10.8)	15 (11.5)	33 (10.5)	
HBV infection before cancer diagnosis	67 (15.1)	14 (10.8)	53 (16.8)	0.104
HCV infection before cancer diagnosis	83 (18.7)	23 (17.7)	60 (19.1)	0.739
AIDS defining illness	289 (64.9)	99 (76.2)	190 (60.3)	0.002
Requiring prophylactic antibiotics (CD4 < 200)	273 (61.4)	94 (72.3)	179 (56.8)	0.003
Median duration from HIV to cancer, years	3.72 (1.49–6.53)	2.88 (1.14–5.96)	4.11 (1.71–6.66)	0.058
**Modified Charlson’s comorbidity score)**				0.231
0	23 (5.2)	5 (3.9)	18 (5.7)	
1–5	373 (83.8)	115 (88.5)	258 (81.9)	
≥ 6	49 (11.0)	10 (7.7)	39 (12.4)	
On ART patients	396 (89.0)	121 (93.1)	275 (87.3)	0.077
Optimal ART adherence (MPR > 95%)	215 (48.3)	55 (42.3)	160 (50.8)	0.103
**Financial status**				0.3678
NHI	405 (91.01)	121 (93.08)	284 (90.16)	
National Medical Aid	40 (8.99)	9 (6.92)	31 (9.84)	

*The date of HIV diagnosis was defined as the date on which HIV-related code was registered in the database.

### Cancer incidence

In total, 445 incident cancer cases were diagnosed between 2004–2017, of which 130 (29.2%) were ADCs and 298 (67.0%) were NADCs (Table 3). NHL (ID: 150.65/100,000 person-years) was the most common ADC in both men and women. Liver cancer (94.82), lung cancer (58.22), colorectal cancer (44.91), and stomach cancer (43.25) were the most common types of cancer in men, whereas breast cancer (101.30) and thyroid cancer (81.04) were the most common types in women.

**Table 3:** Standardized incidence ratios (SIRs) for various cancer types among PLWHA.

Cancer	Male (aged ≥ 15 years; *n *= 10,911)				Female (aged ≥ 15 years; *n* = 826)				Total (aged ≥ 15 years; *N* = 11,737)			
	Person-years: 60,115.89				Person-years: 4935.64				Person-years: 65,051.53			
	case	ID*	SIR	95% CI	case	ID*	SIR	95% CI	case	ID*	SIR	95% CI
Total	406	675.36	1.84	(1.66, 2.03)**	39	790.17	1.61	(1.14, 2.20)**	445	684.07	1.72	(1.57, 1.89)**
ADCs	116	192.96	23.48	(19.40, 28.16)**	14	283.65	10.86	(5.93, 18.22)**	130	199.84	12.64	(10.56, 15.01)**
Kaposi’s sarcoma	25	41.59	415.80	(269.01, 613.84)**	NA	NA	NA	NA	25	38.43	620.89	(401.69, 916.59)**
NHL	91	151.37	18.65	(15.01, 22.89)**	7	141.83	19.78	(7.93, 40.76)**	98	150.65	21.73	(17.64, 26.49)**
Cervix					7	141.83	7.50	(3.00, 15.45)**				
NADCs	290	482.40	1.37	(1.22, 1.54)**	25	506.52	1.11	(0.72, 1.64)	315	484.23	1.30	(1.16, 1.45)**
HPV-related head and neck cancer	9	14.97	1.62	(0.74, 3.08)	2	40.52	15.36	(1.73, 55.46)**	11	16.91	2.97	(1.48, 5.32)**
Stomach	26	43.25	0.63	(0.41, 0.92)**	NA	NA	NA	NA	26	39.97	0.79	(0.51, 1.15)
Colorectal	27	44.91	0.83	(0.55, 1.21)	1	20.26	0.45	(0.01, 2.52)	28	43.04	0.98	(0.65, 1.42)
Anus	19	31.61	85.92	(51.71, 134.18)**	NA	NA	NA	NA	19	29.21	69.52	(41.84, 108.57)**
Liver	57	94.82	2.16	(1.63, 2.79)**	3	60.78	3.77	(0.76, 11.01)	60	92.23	3.31	(2.53, 4.27)**
Pancreas	9	14.97	1.63	(0.74, 3.09)	NA	NA	NA	NA	9	13.84	1.78	(0.81, 3.39)
Lung	35	58.22	1.37	(0.96, 1.91)	3	60.78	2.23	(0.45, 6.53)	38	58.42	1.91	(1.35, 2.63)**
Non-melanoma skin	4	6.65	1.21	(0.33, 3.10)	1	20.26	2.82	(0.04, 15.69)	5	7.69	1.46	(0.47, 3.40)
Breast	1	1.66	6.63	(0.09, 36.88)	5	101.30	1.04	(0.34, 2.43)	6	9.22	0.20	(0.07, 0.44)**
Prostate	25	41.59	1.79	(1.16, 2.64)**								
Kidney and renal pelvis	5	8.32	0.64	(0.21, 1.50)	NA	NA	NA	NA	5	7.69	0.84	(0.27, 1.96)
Bladder	5	8.32	0.99	(0.32, 2.30)	NA	NA	NA	NA	5	7.69	1.53	(0.49, 3.56)
Thyroid	9	14.97	0.46	(0.21, 0.86)**	4	81.04	0.57	(0.15, 1.46)	13	19.98	0.23	(0.12, 0.40)**
Hodgkin’s lymphoma	6	9.98	15.03	(5.49, 32.71)**	1	20.26	55.24	(0.72, 307.34)	7	10.76	20.65	(8.27, 42.55)**
Multiple myeloma	5	8.32	3.81	(1.23, 8.89)**	NA	NA	NA	NA	5	7.69	3.89	(1.25, 9.08)**
Leukemia	5	8.32	1.41	(0.45, 3.29)	NA	NA	NA	NA	5	7.69	1.49	(0.48, 3.48)

*Incidence density per 100,000 person years. **Indicates statistical significance.

Cancer types with fewer than 5 total number of cases are not shown in this table.

ID, incidence density; SIR, standardized incidence ratio; ADCs, AIDS-defining cancers; NADCs, non-AIDS defining cancers; NHL, non-Hodgkin’s lymphoma; CNS, central nervous system.

### SIRs of different cancer types

Overall, cancer risk was 72% higher among PLWHA than among the general population (SIR: 1.72, 95% CI: 1.57–1.89) (Table 3). Compared with the general population, the risk was elevated in those with ADCs (12.64, 10.56–15.01), and the SIR of Kaposi’s sarcoma was markedly increased (620.89, 401.69–916.59), followed by that of NHL (21.51, 17.44–26.24), and cervical cancer (7.5, 3.00–15.45). The risk was significantly elevated for virus-related NADCs, including HPV-related head and neck cancer (2.97, 1.48–5.32), anal cancer (69.52, 41.84–108.57), liver cancer (2.53–4.27), and Hodgkin’s lymphoma (20.65, 8.27–42.55). Risks were also significantly elevated in the following cancer types: lung (1.91, 1.35–2.63), penis (16.97, 1.94–61.28), prostate (1.79, 1.16–2.64), other male genital organs (13.79, 1.55–49.79), eye (18.94, 2.13–68.38), and multiple myeloma (3.89, 1.25–9.08). In contrast, the risk was significantly decreased for cancers of the breast (0.20, 0.07–0.44) and thyroid cancer (0.12–0.40) and stomach cancer in men (0.63, 0.41–0.92).

### Cancer trends among PLWHA

**Figure 1:**
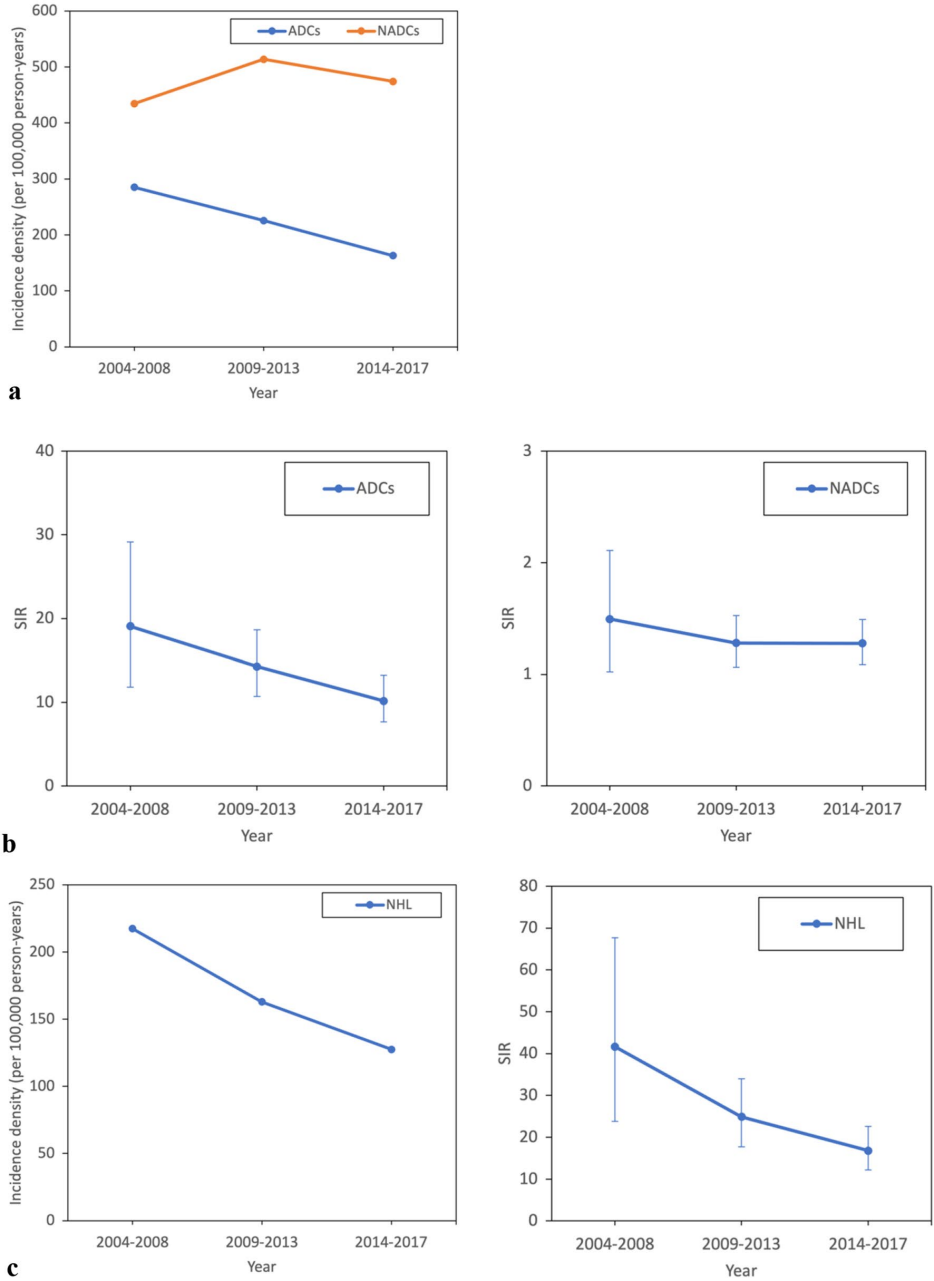

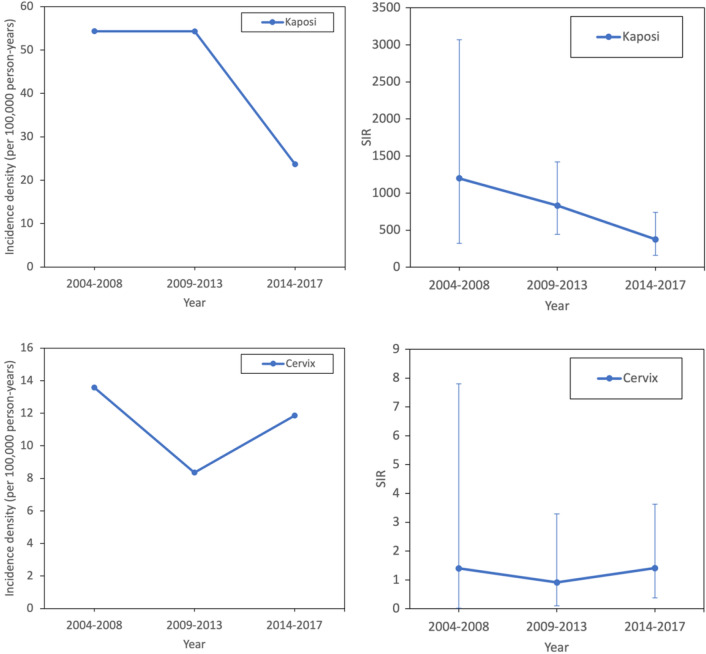
(**a**) Trend of incidence densities (ID) of ADCs and NADCs in 2004–2017 (ADC *p*-value 0.012, NADC *p*-value 0.671), (**b**) trend of standardized incidence ratio (SIR) of ADCs and NADCs in 2004–2017 (ADC *p*-value 0.028, NADC p-value 0.459), and (**c**) trend of ID and SIR of ADC by each cancer. (Kaposi’s sarcoma ID p-value 0.333, SIR *p*-value 0.040; NHL SIR p-value 0.127, NHL ID p-value 0.073; cervical cancer ID *p*-value 0.788, cervical cancer SIR *p*-value 0.989).

From 2004 to 2017, the period-adjusted ADC incidence rate decreased from 285.16 cases/100,000 person-years to 163.00 cases/100,000 person-years (*p* < 0.001) (Fig. 1A). The incidence rate of Kaposi’s sarcoma decreased from 54 cases/100,000 person-years to 20.00 cases/100,000 person-years (*p* = 0.333) (Fig. 1C). The incidence rate of NHL decreased from 271 cases/100,000 person-years to 127 cases/100,000 person-years (*p* = 0.073). However, both decreased incidence rates were not statistically significant. A similar decrease in the SIRs of ADCs was observed (Fig. 1B). The incidence rate and SIR of cervical cancer did not show a decreasing trend. The period-adjusted NADC rate did not show a decreasing trend but was higher than that of the general population during the entire period.

### SIR according to ART adherence

PLWHA with non-optimal ART adherence had significantly higher SIR for some NADCs and ADCs, except for cervical cancer, than those with optimal ART adherence (Table 4). This was observed for each ADC and virus-related NADCs such as anal cancer (SIR ratio: 0.33, 95% CI 0.11–0.90) and liver cancer (0.47, 0.44–0.81). Risks were also higher among PLWHA with non-optimal ART adherence for leukemia.

**Table 4:** Standardized incidence ratio for cancer in PLWHA by ART compliance.

Cancer	SIR (95% CI)		SIR Ratio (95% CI)
	Optimal ART adherence	Non-optimal ART adherence	
Total	1.32 (1.15, 1.51)	2.41 (2.11, 2.75)	0.55 (0.45, 0.66)*
ADCs	8.61 (6.49, 11.21)	19.22 (15.12, 24.09)	0.45 (0.31, 0.64)*
Kaposi’s sarcoma	346.11 (157.93, 657.06)	1,121.90 (640.85, 1822.02)	0.30 (0.12, 0.73)*
NHL	13.98 (9.99, 19.04)	35.18 (26.71, 45.48)	0.40 (0.26, 0.61)*
Cervix	1.71 (0.63, 3.73)	0.45 (0.01, 2.48)	3.84 (0.47, 176.60)
NADCs	0.99 (0.84, 1.16)	1.63 (1.38, 1.92)	0.61 (0.48, 0.77)*
HPV-related head and neck cancer	2.10 (0.68, 4.89)	4.55 (1.66, 9.91)	0.46 (0.11, 1.81)
Stomach	0.71 (0.40, 1.18)	0.91 (0.45, 1.63)	0.78 (0.34, 1.89)
Colorectal	0.88 (0.50, 1.43)	1.17 (0.60, 2.04)	0.75 (0.33, 1.74)
Anus	39.90 (15.98, 82.21)	122.64 (63.30, 214.25)	0.33 (0.11, 0.90)*
Liver	2.35 (1.55, 3.43)	4.97 (3.42, 6.98)	0.47 (0.44, 0.81)*
Pancreas	0.91 (0.18, 2.66)	3.42 (1.25, 7.44)	0.27 (0.04, 1.25)
Lung	1.54 (0.94, 2.38)	2.61 (1.54, 4.12)	0.59 (0.30, 1.19)
Non-melanoma skin	1.79 (0.48, 4.59)	0.83 (0.01, 4.63)	2.16 (0.21, 106.22)
Breast	0.11 (0.01, 0.38)	0.36 (0.10, 0.91)	0.30 (0.03, 2.09)
Prostate	3.18 (1.82, 5.16)	3.63 (1.65, 6.88)	0.88 (0.37, 2.25)
Kidney and renal pelvis	1.31 (0.42, 3.06)	NA (NA)	NA (NA)
Bladder	1.88 (0.51, 4.82)	0.87 (0.01, 4.83)	2.17 (0.22, 106.84)
Thyroid	0.24 (0.10, 0.47)	0.23 (0.07, 0.54)	1.03 (0.30, 4.02)
Hodgkin’s lymphoma	14.36 (2.89, 41.94)	30.78 (8.28, 78.79)	0.47 (0.07, 2.76)
Multiple myeloma	1.19 (0.02, 6.64)	8.95 (2.41, 22.91)	0.13 (0.00, 1.35)
Leukemia	NA (NA)	3.97 (1.28, 9.28)	0.00 (0.00, 0.66)*

Unless otherwise indicated, SIR ratios are from models adjusted for sex, age.

Cancer types with fewer than 5 total number of cases are not shown in this table.

*Indicates statistical significance.

### Overall mortality

**Figure 2:**
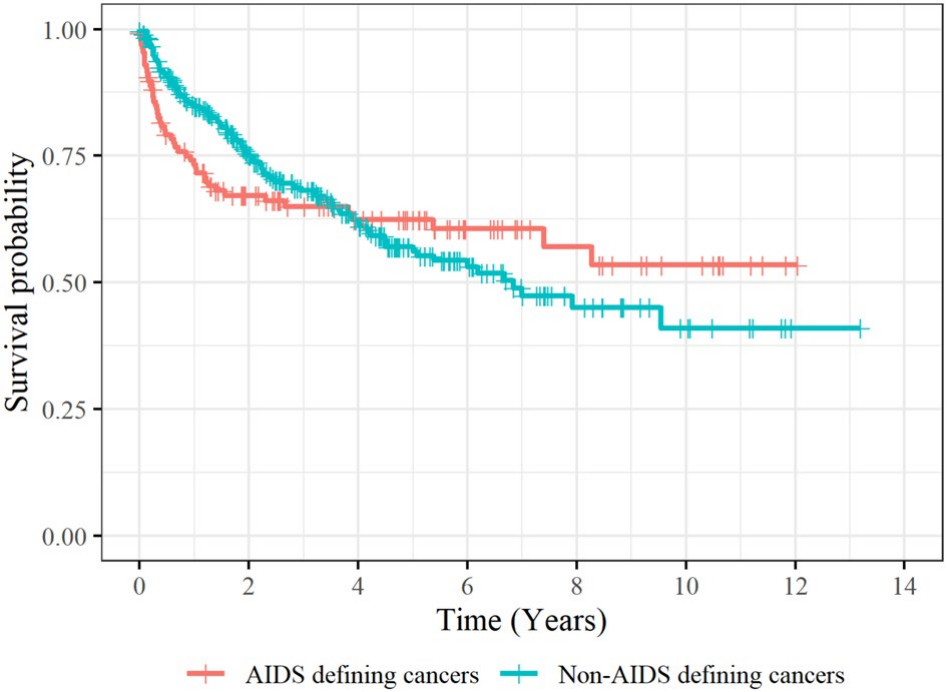
Kaplan–Meier survival curves for PLWHA stratified by cancer type (Wilcoxon *p* = 0.353; Taron-Ware *p* = 0.576).

The 1-year and 5-year survival rates in PLWHA with cancer were 79.9% (ADC: 72.2% vs. NADC: 83.1%) and 57.8% (ADC: 61.5% vs. NADC: 55.9%), respectively (Fig. 2). There was no significant difference between the survival curves of ADC and NADC (*p* = 0.906). There was no significant difference between the survival curves of ADC and NADC (Wilcoxon *p* = 0.353; Taron-Ware *p* = 0.576). In the overall trend, no significant difference was found between the two groups, but when they were analyzed separately for each period, probability of survivial in NADC was significantly higher at the 1st year of cancer onset, and probability of survival in ADC was signifantly higher at 7th and 8th years of onset (Supplementary Table S3).

## Discussion

In this study, a comprehensive analysis of cancer incidence among PLWHA was conducted based on nationwide data from South Korea, with a lengthy follow-up duration and information on mortality. We observed a 1.72-fold increased risk of overall cancer in PLWHA compared with that in the general population. In particular, elevated cancer risk was more prominent in the poor ART adherence group (2.41-fold). The increased risk was noted for various types of cancer but was particularly high for virus-related cancers. Although the incidence of ADCs declined, that of NADCs did not.

Decreasing incidence of ADCs among PLWHA has been noted in Western countries and in Taiwan^11,27–30^. In a study conducted in France from 2010 to 2015, the incidence of ADC was 191 cases/100,000 person-years, the incidence of ADC showed a decreasing trend, and the incidence rate of NADC did not change^28^. In contrast, studies conducted in 2006, 2009, and 2017 in Korea reported no decrease in the incidence rates of ADCs among PLWHA^13,14^. As ART was established and immunity was restored, the incidence rates of Kaposi’s sarcoma and NHL decreased over time, and similarly, the risk of ADC was lower in those with optimal ART adherence. Despite the decreasing cases of ADCs, patients with poor adherence to ART remain vulnerable. A higher SIR was observed for cervical cancer among PLWHA than among the general population; however, because of the small number of cases (*n* = 7), a trend of decreasing incidence over time and association with ART was not confirmed.

During the study period, no significant trends were observed in the incidence or SIR of NADC. Previous studies have reported that, as PLWHA ages, the NADC incidence tends to increase^31–34^. According to the HIV/AIDS notification in Korea by the KCDC, especially since 2010, the number of young adults with HIV infection in their 20 s has rapidly increased^35^. This continuous influx of young adults may have slowed the rise in the cancer incidence rate over time. Some studies have shown that the SIR of NADCs decreases with time^11,27^. Previous studies included all pre-HAART periods, early HAART periods, and late HAART periods; however, this study only included late HAART periods, which may have affected the results.

Park et al. demonstrated that sustained ART, which results in long-term viral suppression, may contribute to cancer prevention, with risk reduction for ADC and virus-related NADC^36^. Additionally, previous studies have shown that enhanced immune status and early initiation of ART could reduce the incidence of virus-related NADCs^11,37^. Similarly, we found that the incidence of ADC and virus-related NADCs was reduced in the optimal ART adherence group, although it remained higher than that in the general population. Therefore, to improve cancer outcomes among PLWHA, adherence to ART and screening for and prevention of virus-related cancers should be promoted. Efforts should also be made to initiate early ART and the maintenance of enhanced immune status.

Risk was elevated for some virus-unrelated NADCs such as lung cancer and prostate cancer in PLWHA, compared with the general population. We found that lung cancer was second only to liver cancer in terms of NADC incidence. Lung cancer patients were not detected in two studies published in 2006 and 2009 among PLWHA in South Korea, whereas in another study published in 2017 lung and liver cancers were among the most diagnosed cancers^12–14^. Lung cancer is the most common cancer type in Korean men aged ≥ 65 years^38^. These results suggest that cancer incidence patterns in Korean PLWHA are gradually becoming similar to those of the general population because of prolonged survival due to the availability of HAART^14^. The risk of lung cancer in PLWHA was elevated relative to that in the general population, which is consistently noted in previous studies^11,39,40^. In addition to the high prevalence of smoking among PLWHA, HIV infection itself, independent of smoking, was associated with an increased risk of lung cancer^41^. Therefore, it is necessary to encourage smoking cessation and participation in screening tests (low-dose computed tomography), which are now included in Korea’s National Cancer Screening Program (NCSP) since 2019.

In contrast to previous findings, we found a higher incidence of prostate cancer among PLWHA than among the general population^11,31,42^. In Korea, prostate cancer incidence has increased significantly compared to that in the past, partly due to the rise in average life expectancy, westernized dietary habits, and increased awareness of prostate cancer screening^43,44^. The genitourinary tract is a common vector for HIV transmission, and HIV status is a risk factor for lower urinary tract symptoms^45^. This may explain the high SIR of prostate cancer as PLWHA are more likely to visit the hospital regularly and get tested if they have urinary infection symptoms. Similarly, a study conducted in Taiwan has reported an increase in the SIR of prostate cancer^27^.

Consistent with previous studies, an increase in SIR was not observed for most virus-unrelated NADC, and a decreased in SIR was observed in some cases such as stomach cancer, thyroid cancer, breast cancer. The low risk of stomach and thyroid gland cancers among PLWHA may be because of decreased surveillance. In Korea, screening for stomach, liver, colorectal, breast, cervical, and lung cancers are included in the NCSP, and that for thyroid cancer is widely available^46,47^. In the general Korean population, the screening rate with recommendations for stomach cancer has consistently been > 70% since 2012^48^. Vulnerable populations, such as those with low income, low education level, and unhealthy behaviors (alcohol consumption), were associated with lower participation in gastric cancer screening programs in South Korea^49^. Thus, compared with the general population, PLWHA may have an underestimated risk of cancer, especially those widely screened, owing to poor screening. In contrast, in a study of South Korean patients who received solid organ transplantation, the SIRs for thyroid, stomach, and breast cancers increased compared with those reported in the general population, which may be explained by increased surveillance rate^50^. Although the 5-year survival rate of cancer in Korea was 70.3%, that of PLWHA with NADCs was only 55.9%, suggesting that cancers are diagnosed at a more advanced stage among PLWHA than among the general population. Therefore, cancer screening rates should be increased for the early detection of cancer among PLWHA.

No significant differences were found in overall survival between patients with ADC and patients with NADC, but there were differences in some time periods. During the first year, the survival rate of patients with ADC decreased sharply, but after one year, the survival rate did not decrease significantly. However, the survival rate of patients with NADC continued to decrease consistently for eight years. The lower survival rate in patients with ADC during the first year may be due to advanced immunodeficiency. In our study, patient with ADC were diagnosed with cancer at a younger age than patient with NADC, but they were more immunocompromised. In a previous study conducted in Korea, in spite of survival improvement in the HAART era, the early mortality was still substantial in late-HAART period, presumbably because the patients were in advanced immunodeficiency at initial presentation^51^. In addition, this immunodeficiency may have delayed the treatment of cancer, suggesting that the early initiation of ART is also important for cancer treatment. After 7 years, the survival rate of NADC interestingly showed a decrease, which may be attributed to the adverse effects of cancer treatment. When ART and antineoplastic agent are combined, enhanced and overlapping toxics effect are possible^52^. One study demonstrated that immunosuppression in PLWHA driven by cancer treatment, such as chemotherapy and radiotherapy, can result in an increased risk of mortality^53^. A cancer treatment strategy, based on the characteristics of HIV patients, is therefore warranted.

This study had several limitations. First, HIV/AIDS and cancer incidence rates were identified through the medical insurance code and the ICD-10 code in the NHISS claims data, with potential misclassification. To address this limitation, we only included HIV/AIDS cases that had been diagnosed using the relevant diagnostic code, wherein the HIV-related test code had been prescribed, and hospitalization or at least three outpatient visits were recorded. Moreover, we confirmed that most patients (95% in PLWHA) met the RID criteria. The identification of cancer cases based on claims data is considered reliable in Korea because cancer codes are reviewed by the NHIS and provide additional benefits to patients^22^. Therefore, we believe that the definitions of PLWHA and patients with cancer used in this study are reliable. Second, although we standardized age and stratified by sex to eliminate their effect on cancer incidence between PLWHA and the general population, other risk factors of cancer, such as smoking, alcohol use, and the prevalence of oncogenic viral infection, were not considered, owing to a lack of information. Third, as we had extracted data from the data from the database in 2019, we were able to retrieve data only a recent as 2017, therefore we defined our search period as between 2005 to 2017; but the trend of NADC was not clear, and the number of cancers was not sufficient to analyze the tendency for each cancer, so a study including an additional period may be necessary. Finally, as the claim data did not include clinical data in addition to laboratory test results, we were unable to extract data on CD4 count and HIV viral load, which could have helped predict the level of immune dysfunction. Therefore, we used prophylactic antibiotics and AIDS-defining illnesses as indicators of advanced immune suppression.

Overall, although cancer risk has declined in PLWHA in South Korea, the risks for ADCs and many other virus-related cancers remain. We found that cancer incidence in patients with optimal compliance with ART decreased but remained higher than that in the general population. Our findings suggest that close surveillance and routine screening of cancers and improvement in adherence to ART are required to improve the clinical outcomes of PLWHA.

## Supplementary Information

Supplementary Information.

## Data Availability

The datasets generated during and/or analyzed during the current study are not publicly available due to restrictions by Korean NHIS. However, interested parties may submit a separate application to the NHIS for access. The NHIS accepts applications via their website (https://nhiss.nhis.or.kr), and ethics approval from the researcher’s institutional review board and a study proposal are required.
