# Supplementary material for: Nationwide population-based incidence of cancer among patients with HIV/AIDS in South Korea
Source: Sci Rep. 2022 Jun 15;12:9974. doi: 10.1038/s41598-022-14170-5 (PMC9200856; doi:10.1038/s41598-022-14170-5)
Supplement: Supplementary file 1 — Supplementary Information. [file 41598_2022_14170_MOESM1_ESM.docx]

**Nationwide population-based incidence of cancer among patients with HIV/AIDS in South Korea**

Soon Ok Lee^1^, Jeong Eun Lee^1,^ Shinwon Lee^1^, Sun Hee Lee^1*^, Jin Suk Kang^2^, Hyunjin Son^3^, Hyungi Lee^4^, Jinmi Kim^4^

Supplementary Table S1. Operational definitions of cancer

| **Variable of interest** | **Operational definition**  The presence of the following C code more than three times within a year or inpatient hospitalization with a C code |
| --- | --- |
| **AIDS defining cancers** | |
| Kaposi’s sarcoma | Diagnosis records (C46) in inpatient or outpatient file |
| Non-Hodgkin’s lymphoma | Diagnosis records (C82-86, C96) in inpatient or outpatient file |
| Cervix | Diagnosis records (C53) in inpatient or outpatient file |
| **Non-AIDS defining cancers** | |
| Lip | Diagnosis records (C00) in inpatient or outpatient file |
| HPV-related head and neck cancer | Diagnosis records (C01-06, C09-10, C12-14, C32) in inpatient or outpatient file |
| Salivary gland cancer | Diagnosis records (C07-08) in inpatient or outpatient file |
| Nasopharynx | Diagnosis records (C11) in inpatient or outpatient file |
| Stomach | Diagnosis records (C16) in inpatient or outpatient file |
| Colorectal | Diagnosis records (C18-20) in inpatient or outpatient file |
| Anus | Diagnosis records (C21) in inpatient or outpatient file |
| Liver | Diagnosis records (C22) in inpatient or outpatient file |
| Gallbladder etc. | Diagnosis records (C23-24) in inpatient or outpatient file |
| Pancreas | Diagnosis records (C25) in inpatient or outpatient file |
| Lung | Diagnosis records (C33-34) in inpatient or outpatient file |
| Bone | Diagnosis records (C40-41) in inpatient or outpatient file |
| Melanoma | Diagnosis records (C43) in inpatient or outpatient file |
| Non-melanoma skin | Diagnosis records (C44) in inpatient or outpatient file |
| Mesothelioma | Diagnosis records (C45) in inpatient or outpatient file |
| Connective and soft tissue | Diagnosis records (C47, C49) in inpatient or outpatient file |
| Breast | Diagnosis records (C50) in inpatient or outpatient file |
| Ovary | Diagnosis records (C56) in inpatient or outpatient file |
| Penis | Diagnosis records (C60) in inpatient or outpatient file |
| Prostate | Diagnosis records (C61) in inpatient or outpatient file |
| Other male genital organs | Diagnosis records (C63) in inpatient or outpatient file |
| Kidney and renal pelvis | Diagnosis records (C64-65) in inpatient or outpatient file |
| Ureter | Diagnosis records (C66) in inpatient or outpatient file |
| Bladder | Diagnosis records (C67) in inpatient or outpatient file |
| Eye | Diagnosis records (C69) in inpatient or outpatient file |
| Brain, Central nerve system | Diagnosis records (C70-72) in inpatient or outpatient file |
| Thyroid | Diagnosis records (C73) in inpatient or outpatient file |
| Hodgkin’s lymphoma | Diagnosis records (C81) in inpatient or outpatient file |
| Multiple myeloma | Diagnosis records (C90) in inpatient or outpatient file |
| Leukemia | Diagnosis records (C91-95) in inpatient or outpatient file |

Supplementary Table S2. Operational definitions of comorbidities

| **Variable of interest** | **Operational definition** |
| --- | --- |
| **Comorbidities (according to Charlson’s Comorbidity Index [CCI])** | |
| Myocardial infarction (CCI score 1) | Diagnosis records (I21.x, I22.x, I25.2) in inpatient or outpatient file |
| Congestive heart failure (1) | Diagnosis records (I09.9, I11.0, I13.0, I13.2, I25.5, I42.0, I42.5–I42.9, I43.x, I50.x, P29.0) in inpatient or outpatient file |
| Peripheral vascular disease (1) | Diagnosis records (I70.x, I71.x, I73.1, I73.8, I73.9, I77.1, I79.0, I79.2, K55.1, K55.8, K55.9, Z95.8, Z95.9) in inpatient or outpatient file |
| Cardiovascular disease (1) | Diagnosis records (G45.x, G46.x, H34.0, I60.x-I69.x) in inpatient or outpatient file |
| Dementia (1) | Diagnosis records (F00.x–F03.x, F05.1, G30.x, G31.1) in inpatient or outpatient file |
| Chronic pulmonary disease (1) | Diagnosis records (I27.8, I27.9, J40.x–J47.x, J60.x–J67.x, J68.4, J70.1, J70.3) in inpatient or outpatient file |
| Rheumatic disease (1) | Diagnosis records (M05.x, M06.x, M31.5, M32.x-M34.x, M35.1, M35.3, M36.0) in inpatient or outpatient file |
| Peptic ulcer disease (1) | Diagnosis records (K25.x-K28.x) in inpatient or outpatient file |
| Mild liver disease (1) | Diagnosis records (B18.x, K70.0-K70.3, K70.9, K71.3–K71.5, K71.7, K73.x, K74.x, K76.0, K76.2–K76.4, K76.8, K76.9, Z94.4) in inpatient or outpatient file |
| Diabetes without chronic complication (1) | Diagnosis records (E10.0, E10.1, E10.6, E10.8, E10.9, E11.0, E11.1, E11.6, E11.8, E11.9, E12.0, E12.1, E12.6, E12.8, E12.9, E13.0, E13.1, E13.6, E13.8, E13.9, E14.0, E14.1, E14.6, E14.8, E14.9) in inpatient or outpatient file |
| Diabetes with chronic complication (2) | Diagnosis records (E10.2–E10.5, E10.7, E11.2–E11.5, E11.7, E12.2–E12.5, E12.7, E13.2–E13.5, E13.7, E14.2-E14.5, E14.7) in inpatient or outpatient file |
| Hemiplegia or paraplegia (2) | Diagnosis records (G04.1, G11.4, G80.1, G80.2, G81.x, G82.x, G83.0-G83.4, G83.9) in inpatient or outpatient file |
| Renal disease (2) | Diagnosis records (I12.0, I13.1, N03.2–N03.7, N05.2– N05.7, N18.x, N19.x, N25.0, Z49.0–Z49.2, Z94.0, Z99.2) in inpatient or outpatient file |
| Moderate or severe liver disease (3) | Diagnosis records (I85.0, I85.9, I86.4, I98.2, K70.4, K71.1, K72.1, K72.9, K76.5, K76.6, K76.7) in inpatient or outpatient file |
| **AIDS-defining illness (ADIs)*** | |
| Candidiasis of the esophagus, bronchi, trachea, or lungs | Diagnosis records (B371, B3781) in inpatient or outpatient file |
| Coccidioidomycosis, disseminated or extrapulmonary | Diagnosis records (B383, B384, B387, B388) in inpatient or outpatient file |
| Cryptococcosis, extrapulmonary | Diagnosis records (B451, B452, B453, B457, B458) in inpatient or outpatient file |
| Cryptosporidiosis, chronic intestinal (>1-month duration) | Diagnosis records (A072) in inpatient or outpatient file (There is condition of duration of more than a month, but the diagnosis does not reflect this.) |
| Cytomegalovirus disease or CMV (other than liver, spleen, or nodes) | Diagnosis records (B250, B258) in inpatient or outpatient file |
| Cytomegalovirus retinitis (with loss of vision) | No specific diagnostic code |
| Encephalopathy, HIV related  Encephalopathy attributed to HIV | Diagnosis records (B220) in inpatient or outpatient file |
| Progressive multifocal leukoencephalopathy | Diagnosis records (A812) in inpatient or outpatient file |
| Herpes simplex: chronic ulcer(s) (>1-month duration); or bronchitis, pneumonitis, or esophagitis | Diagnosis records (B0089, B009) in inpatient or outpatient file (There is condition of duration of more than a month, but the diagnosis does not reflect this.) |
| Histoplasmosis, disseminated or extrapulmonary | Diagnosis records (B393, B394) in inpatient or outpatient file |
| Isosporiasis, chronic intestinal (>1-month duration) | Diagnosis records (A073) in inpatient or outpatient file (There is condition of duration of more than a month, but the diagnosis does not reflect this) |
| *Mycobacterium avium* complex or *M kansasii,* disseminated or extrapulmonary – disseminated or extrapulmonary infection | No specific diagnostic code |
| *Mycobacterium tuberculosis,* any site (pulmonary or extrapulmonary) | Diagnosis records (A15, A16, A17, A18, A19, P370, J65, B90, M011, M490, M900, N330, N740, N741, K930, K673, K230, O980, U843) in inpatient or outpatient file |
| *Mycobacterium*, other species or unidentified species, disseminated or extrapulmonary | No specific diagnostic code |
| Pneumocystis pneumonia (PCP) | Diagnosis records (B206, B59) in inpatient or outpatient file |
| Pneumonia, recurrent | At least 2 diagnosis records (J10–J18) in inpatient or outpatient file in one year |
| *Salmonella* septicemia, recurrent | At least 2 diagnosis records (A021) in inpatient or outpatient file in one year |
| Toxoplasmosis of brain | Diagnosis records (B582) in inpatient or outpatient file (There is condition of duration of more than a month, but the diagnosis does not reflect this.) |
| Wasting syndrome due to HIV | Diagnosis records (B222) in inpatient or outpatient file |

*The period between two independent event courses for other ADIs was >30 days.

Total number of Korean patients with HIV from 2002–2017

(n = 37,143)

Foreigners (n = 920) and patients with unknown age and sex (n = 142) were excluded.

Korean patients with HIV from 2002–2017 (n = 14,280)

Patients who never received any follow-up tests, including HIV RNA test, T cell subset analysis, and HIV drug resistance mutation sequencing, were excluded (n = 22,863).

Korean patients with HIV from 2002–2017 (n = 13,813)

Patients diagnosed with any type of cancer before the diagnosis of HIV (n = 448) or within the first 3 months after the diagnosis of HIV (n= 290) were excluded.

Incident patients with HIV from 2004–2017 in Korea (n = 12,515)

Deselected patient with HIV related code from 2002 to 2003 (n = 1,244)

and those aged <15 years (n= 54) were excluded.

Cancer-free Korean patients with

(n = 11,737)

Patients with less than three outpatient visits and no hospitalisation were excluded (n = 467).

Patients with at least one HIV-related diagnosis (B20–B24, O987, Z21, R75, V103) from 2002–2016

(N = 38,205)

Supplementary figure S1. PLWHA selection flowchart

Supplementary Table S3. Probability of survival at each time point for PLWHA stratified by cancer type

| AIDS defining cancers | | | | | | | Non-AIDS defining cancers | | | | | | |  | |  | |  | |
| --- | --- | --- | --- | --- | --- | --- | --- | --- | --- | --- | --- | --- | --- | --- | --- | --- | --- | --- | --- |
| Time (Years) | Number at Risk | Number of Event | Survival Probability | SE | 95% CI | | Time (Years) | Number at Risk | Number of Event | Survival Probability | SE | 95% CI | | | Difference of Survival | | p-value | |  |
|  |  |  |  |  | Lower | Upper |  |  |  |  |  | Lower | Upper | |  |  |  |  |  |
| 0 | 130 | 1 | 0.992 | 0.008 | 0.977 | 1.000 | 0 | 315 | 1 | 0.997 | 0.003 | 0.991 | 1.000 | | 0.005 | |  | |  |
| 1 | 87 | 32 | 0.733 | 0.040 | 0.659 | 0.815 | 1 | 234 | 44 | 0.847 | 0.021 | 0.807 | 0.890 | | 0.114 | | * | |  |
| 2 | 67 | 7 | 0.672 | 0.043 | 0.593 | 0.761 | 2 | 172 | 24 | 0.751 | 0.026 | 0.701 | 0.804 | | 0.079 | | NS | |  |
| 3 | 56 | 2 | 0.650 | 0.044 | 0.569 | 0.742 | 3 | 135 | 15 | 0.681 | 0.029 | 0.626 | 0.741 | | 0.031 | | NS | |  |
| 4 | 46 | 2 | 0.624 | 0.046 | 0.540 | 0.721 | 4 | 99 | 11 | 0.618 | 0.032 | 0.557 | 0.684 | | -0.006 | | NS | |  |
| 5 | 39 | 0 | 0.624 | 0.046 | 0.540 | 0.721 | 5 | 65 | 7 | 0.570 | 0.034 | 0.506 | 0.642 | | -0.054 | | NS | |  |
| 6 | 25 | 1 | 0.606 | 0.048 | 0.518 | 0.708 | 6 | 46 | 4 | 0.531 | 0.037 | 0.463 | 0.610 | | -0.075 | | NS | |  |
| 7 | 18 | 0 | 0.606 | 0.048 | 0.518 | 0.708 | 7 | 30 | 4 | 0.474 | 0.043 | 0.396 | 0.566 | | -0.132 | | * | |  |
| 8 | 16 | 1 | 0.570 | 0.057 | 0.469 | 0.694 | 8 | 20 | 1 | 0.451 | 0.047 | 0.368 | 0.552 | | -0.119 | | * | |  |
| 9 | 12 | 1 | 0.535 | 0.064 | 0.423 | 0.675 | 9 | 13 | 0 | 0.451 | 0.047 | 0.368 | 0.552 | | -0.084 | | NS | |  |
| 10 | 9 | 0 | 0.535 | 0.064 | 0.423 | 0.675 | 10 | 9 | 1 | 0.410 | 0.058 | 0.311 | 0.540 | | -0.125 | | NS | |  |
| 11 | 4 | 0 | 0.535 | 0.064 | 0.423 | 0.675 | 11 | 6 | 0 | 0.410 | 0.058 | 0.311 | 0.540 | | -0.125 | | NS | |  |
| 12 | 1 | 0 | 0.535 | 0.064 | 0.423 | 0.675 | 12 | 1 | 0 | 0.410 | 0.058 | 0.311 | 0.540 | | -0.125 | | NS | |  |
|  |  |  |  |  |  |  | 13 | 1 | 0 | 0.410 | 0.058 | 0.311 | 0.540 | |  | |  | |  |

Log-rank p=0.861; Wilcoxon p=0.353; Taron-Ware p=0.576
* p<0.05; NS: Not Significant; SE: Standard Error
